# Profiles of healthcare use of persons living with dementia: A population‐based cohort study

**DOI:** 10.1111/ggi.14930

**Published:** 2024-07-05

**Authors:** Isabelle Dufour, Eva Margo‐Dermer, Catherine Hudon, Caroline Sirois, Claire Godard‐Sebillotte, Nadia Sourial, Louis Rochette, Amélie Quesnel‐Vallée, Isabelle Vedel

**Affiliations:** ^1^ School of Nursing, Faculty of medicine and health sciences Université de Sherbrooke Sherbrooke Quebec Canada; ^2^ Research Center of Aging Université de Sherbrooke Sherbrooke Quebec Canada; ^3^ Department of Family Medicine, Faculty of Medicine and Health Sciences, Faculty of Medicine McGill University Montreal Quebec Canada; ^4^ Department of Family Medicine and Emergency medicine, Faculty of medicine and health sciences Université de Sherbrooke Sherbrooke Quebec Canada; ^5^ Faculty of Pharmacy Université Laval Quebec City Quebec Canada; ^6^ Department of Medicine Division of Geriatrics McGill University Montreal Quebec Canada; ^7^ McGill University Health Centre (MUHC) Research Institute Montreal Quebec Canada; ^8^ Department of Health Management, Evaluation and Policy; School of Public Health University of Montreal Montréal Quebec Canada; ^9^ National Public Health Institute of Québec Quebec City Quebec Canada; ^10^ Department of Epidemiology, Biostatistics, and Occupational Health, Faculty of Medicine McGill University Montreal Quebec Canada; ^11^ Department of Sociology, Faculty of Arts McGill University Montreal Quebec Canada; ^12^ Lady Davis Institute for Medical Research Jewish General Hospital Montréal Quebec Canada

**Keywords:** aged, classification, delivery of health care, dementia, latent class analysis

## Abstract

**Aim:**

Persons living with dementia are a heterogeneous population with complex needs whose healthcare use varies widely. This study aimed to identify the healthcare use profiles in a cohort of persons with incident dementia, and to describe their characteristics.

**Methods:**

This is a retrospective cohort study of health administrative data in Quebec (Canada). The study population included persons who: (i) had an incident dementia diagnosis between 1 April 2015 and 31 March 2016; (ii) were aged ≥65 years and living in the community at the time of diagnosis. We carried out a latent class analysis to identify subgroups of healthcare users. The final number of groups was chosen based on clinical interpretation and statistical indicators.

**Results:**

The study cohort consisted of 15 584 individuals with incident dementia. Four profiles of healthcare users were identified: (i) Low Users (36.4%), composed of individuals with minimal healthcare use and fewer comorbidities; (ii) Ambulatory Care‐Centric Users (27.5%), mainly composed of men with the highest probability of visiting cognition specialists; (iii) High Acute Hospital Users (23.6%), comprised of individuals mainly diagnosed during hospitalization, with higher comorbidities and mortality rate; and (iv) Long‐Term Care Destined Users (12.5%), who showed the highest proportion of antipsychotics prescriptions and delayed hospitalization discharge.

**Conclusions:**

We identified four distinct subgroups of healthcare users within a population of persons living with dementia, providing a valuable context for the development of interventions tailored to specific needs within this diverse population. **Geriatr Gerontol Int 2024; 24: 789–796**.

## Graphical abstract

Based on a cohort of 15 584 persons with incident dementia, we identified four healthcare use profiles: (i) Low Users (minimal healthcare needs); (ii) Ambulatory Care users (frequent cognition specialist visits); (iii) High Acute Hospital users (high hospitalization rates); and (iv) Long‐Term Care users (antipsychotic use and delayed hospital discharge).

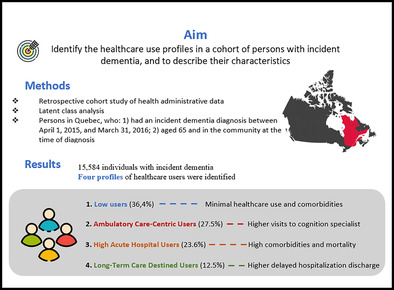

## Introduction

Population aging is a pressing global issue, leading to the increased prevalence of dementia, a recognized health priority.^1^ In Canada, the number of persons living with dementia (PLWD) is projected to triple, to exceed 950 000 by 2030.^2^ Dementia is a neurocognitive disorder that can significantly affect daily functioning and cause distress not only for those diagnosed with the condition, but also for their loved ones and caregivers.^3^

The complex nature of this condition makes its management complex, notably due to dementia severity, behavioral and psychological symptoms of dementia, challenges with managing comorbid conditions, and polypharmacy.^4^ In contrast, healthcare systems face challenges in meeting the growing demands of dementia care and associated healthcare needs.^3^, ^5^, ^6^ Despite notable improvements over the recent years, many barriers to optimal care remain, particularly at the health system level, including complexity and accessibility of services, fragmentation of care, and lack of coordinated care and professional collaboration.^3^, ^5^ At the individual level, attitudes, beliefs and social environment also have an impact on services allocation.^7^, ^8^

PLWD show clear patterns of higher healthcare use compared with their same‐age counterparts without dementia. Notably, PLWD are more likely to visit the emergency department (ED) and approximately 15% are high users of these settings.^9^, ^10^ Their hospitalization rate is approximately 65% higher, their length of stay is nearly twice as long and they are prone to delayed discharge or hospitalization for preventable reasons.^9^, ^10^ Approximately 40% of PLWD will experience a transfer to long‐term care facilities (LTCF).^9^ They also use higher rates of home care services and visit their family physician more frequently than their counterparts without dementia.^10^ Studies reported that healthcare needs and care transitions of PLWD are higher in the year after the dementia diagnosis.^11^, ^12^ Healthcare use is also increased during this period, with higher primary care and specialist visits, and hospitalizations.^13^ This period could be a strategic target for interventions, such as case management, as it represents a period of heightened vulnerability.^12^, ^14^

However, PLWD represents a heterogeneous population, including patients with very different needs and vulnerability factors, and thus with diverse healthcare use patterns. Furthermore, we know even less about the heterogeneity of newly diagnosed individuals, representing a crucial moment in the disease trajectory marked by significant healthcare use.^12^ Understanding these variations is thus a crucial step toward the delivery of targeted interventions. Using classification methods, previous studies on small cohorts aimed to improve our knowledge on healthcare use for PLWD.^15^, ^16^, ^17^ These studies have revealed the co‐occurrence of specific characteristics, and highlighted the unique needs of each group. Although they provided valuable insight on the diversity of PLWD needs and healthcare use patterns, there is still a lack of research on province‐wide populations, which might offer a more comprehensive portrait of healthcare use. Also, examining healthcare use patterns in the year after a dementia diagnosis would bring additional valuable information on this critical period when PLWD are taken care of by the healthcare system and oriented to diverse resources. Thus, the present study aimed to develop profiles of healthcare use among PLWD in the year after a first dementia diagnosis in a population‐based cohort.

## Methods

### Design and data sources

The present retrospective cohort study used the Quebec Integrated Chronic Disease Surveillance System held at the Institut national de santé publique du Québec (The National Public Health Institute of Quebec).^18^ This database comprises health services records provided by the provincial health insurance board (Régie de l'assurance maladie du Québec), which provides universal health insurance coverage to all residents (~8 000 000 inhabitants) of the Province of Quebec (Canada).^18^ The covered services include those provided in ED, hospitals, outpatient and primary care clinics, home care services, and accommodation in public long‐term care centers for eligible people.^19^

The Quebec Integrated Chronic Disease Surveillance System notably gives access to these datasets: (i) patient demographic information (e.g. age, sex, region of residence); (ii) medical consultations (data on medical services provided by Quebec fee‐for‐service physicians, including diagnoses coded according mostly to the International Classification of Diseases 9th revision); (iii) drugs (data on pharmacy‐claimed drugs, for patients covered by the public drug insurance plan – coverage of approximately 90% of Quebec's population aged ≥65 years, excluding those in LTCF); and (iv) hospitalizations (information on hospitalization, including diagnosis coded in the International Classification of Diseases 10th revision).^18^

### Studied population

The study population included individuals from Quebec (Canada) who: (i) had an incident dementia diagnosis between 1 April 2015 and 31 March 2016; and (ii) were aged >65 years, and community‐dwelling on diagnosis. The index date is the date of the diagnosis. We considered an individual as community‐dwelling if the person was not living in a LTCF.^20^ We identified dementia diagnosis using a validated algorithm.^21^ Individuals were considered to have a dementia diagnosis if they met at least one of the following three criteria: (i) H criterion: one diagnosis code for dementia in hospitalization dataset; (ii) M criterion: three dementia diagnoses in the medical consultation dataset registered at least 30 days apart in 2 years; and (iii) R criterion: a prescription claimed for a dementia‐related drug in the drug dataset. The date of dementia identification was when the first of the three criteria became positive.

### Variables

#### Sociodemographic variables

First, we considered the following variables at the index date: age, sex, the cohort inclusion criterion (related to which became positive first in our dementia algorithm; whether the patient was included in the cohort based on H, M or R criterion)^21^ and the Pampalon material deprivation index based on geographic area of residence (quintile 1 – least deprived – to quintile 5 – most deprived).^22^ Second, we considered the following variables in the 2 years preceding the index date: comorbidity index (excluding dementia),^23^ physical and psychological comorbidities, based on at least one of these criteria: (i) one diagnosis code for the condition in the hospitalization dataset; and (ii) two diagnosis codes for the condition in the medical consultation dataset.^23^

#### Healthcare use variables

The following variables were considered 1 year after the index date (incident diagnosis of dementia): (i) number of family physician visits (≥1 visits, ≥8 visits); (ii) number of cognition specialist visits (geriatricians, neurologist, psychiatrist; dichotomized: 0, ≥1 visits); (iii) number of other specialist visits (dichotomized: 0, ≥1 visits); (iv) number of ED visits (≥1 visits, ≥4 visits); (v) number of days in the hospital; (vi) 30‐day hospital readmission (yes/no); (vii) LTCF admission (i.e. permanent relocation to a long‐term care facility with nursing care 24/7 [yes/no]); (viii) mortality (yes/no); and (ix) number of days in delayed hospital discharge defined as alternate level of care in the database (dichotomized: 0, ≥1 days). Delayed hospital discharge (alternate level of care) describes a situation where patients are in a condition to be discharged from the hospital, but cannot return to independent living and are mostly waiting for a LTCF bed to become available.^24^ Regarding medication, we considered the following variables in the year after the index date: total number of different medications and use of medication that are specific to dementia treatment: acetylcholinesterase inhibitor or memantine, antipsychotic, benzodiazepine and antidepressant (yes/no for each medication).

### Statistical analysis

First, descriptive statistics were computed to describe the overall characteristics of the sample. Second, we used a latent class analysis (LCA) approach to identify the profiles of healthcare use for PLWD.^25^, ^26^ LCA is an model‐based approach that identifies heterogeneity by analyzing individual patterns of behavior for common types of subgroups or classes. Estimation relies on observed individual characteristics, referred to as indicators, which enable the classification of homogeneous groups where individuals in one group are similar and distinct from those in other groups. The probabilistic nature of LCA allows for comparing solutions using statistical criteria, and accommodates continuous and categorical variables.

The most relevant indicators for differentiating the group structure in the data were retained for the final model. The indicators and covariates were selected based on scientific literature and clinical expertise of the authors (nursing, geriatric, community pharmacy, family medicine and public health). Our choices ensured the interpretability and pertinence of the final profiles. The final model included the nine aforementioned variables on healthcare use as indicators, as well as covariates known to impact healthcare use: age, sex, material deprivation index, comorbidity index and cohort inclusion criterion (H, M, R).

In the initial step of the analyses, a series of increasingly complex models (adding classes from 1 to 5) were estimated to determine the optimal number of latent classes. Following standard practice,^27^ Akaike and Bayesian Information Criteria were used for selecting the final analytical classification model (See Appendix I in Table A1). Although numerous statistical criteria for class separation exist, Bayesian Information Criteria is deemed superior,^28^, ^29^ and a combination of Akaike and Bayesian Information Criteria has been recommended.^30^ The final selection of the number of classes was based on a balance between achieving optimal statistical criteria and maintaining clinical significance, by relaying on the experience of the various methodological experts and healthcare professionals involved in the study.^28^, ^31^ For the final model, the probabilities of group membership were computed for each individual. An individual was then assigned to the group with the highest probability. Variables within the final latent classes were compared using the χ^2^‐test (for categorical variables) and Kruskal–Wallis test (for continuous variables) for significance. In coherence with the method, we chose to present the LCA class membership and probabilities (which represent how likely patients in one given class are to provide different values on indicators) for the final model. It should be noted that mortality was excluded for all persons in the cohort admitted to LTCF in the LCA analysis, as healthcare use was not measured after institutionalization due to data limitations. Thus, all the reported variables (except mortality rate) were considered before LCTF admission. Finally, the variables within the groups composing the final latent classes were compared using χ^2^‐test (for categorical variables) and Kruskal–Wallis test (for continuous variables) for significance, with a significance level of 0.001. All analyses were carried out by EMD with the R Software Version 3.5.1 using the poLCA package (The R Foundation for Statistical Computing, Vienna, Austria).^32^

## Results

### Study cohort description

The cohort was comprised of 15 584 individuals, with 60.6% women and over half (62.5%) aged ≥80 years (median 82 years; range 65–106 years). Regarding the dementia diagnosis, approximately half of the cohort (*n* = 7540; 48.4%) were identified by the M criterion (diagnosis from medical consultations dataset), 3192 individuals (20.5%) were identified based on the H criterion (diagnosis in hospitalization dataset) and 4852 (31.1%) on the R criteria (dementia‐related drug in drug dataset). In the year after diagnosis, 2173 persons (13.9%) were admitted to LTCF and 1611 (10.4%) died (Table 1).

**Table 1:** Characteristics of the overall cohort (*n* = 15 584)

Variables	
Median age, years (range)	82 (65–106)
Female, *n* (%)	9443 (60.6)
Material deprivation, *n* (%)	
1–2 (least deprived)	4191 (29.9)
3	2617 (16.8)
4–5 (most deprived)	5649 (36.3)
Cohort inclusion criterion, *n* (%)^†^	
H criterion	3192 (20.5)
M Criterion	7540 (48.4)
R criterion	4852 (31.1)
LTCF admissions during follow up, *n* (%)	2173 (13.9)
Survival at 1 year, *n* (%)	13 973 (89.7)
Death, *n* (%)	
Outside LTCF	1177 (7.6)
In LTCF	434 (2.8)

Abbreviation: LTCF, long‐term care facility.

^†^ H criterion: one hospitalization with dementia code; M criterion: three physician visits with dementia codes; R criterion: prescription profile consistent with dementia.

### Latent class solution

**Figure 1:**
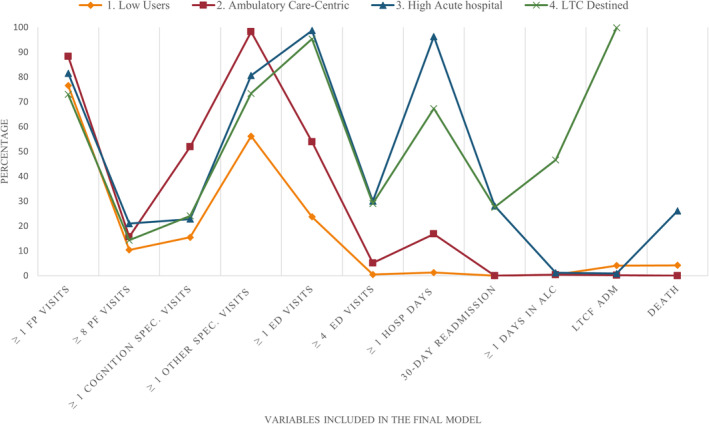
Probabilities, given class membership, for each class within the final four class model. ALC, alternate level of care; ED, emergency department; FP, family physicians; Hosp, hospitalization; LTCF ADM, long‐term care facility admission; Spec, specialists.

The four‐class model was retained as the optimal classification in terms of fit and relevance (see Appendix I in Table A1 for statistical indicators results for increasing numbers of classes). Table 2 and Figure 1 respectively provide LCA class membership and probabilities for the final model, and its visual representation. Table 3 shows the characteristics of individuals per group.

**Table 2:** Latent class analysis class membership and probabilities for the final model

Variables	Group 1: low users	Group 2: ambulatory care‐centric users	Group 3: high acute hospital users	Group 4: long‐term care destined users
	*n* = 5673 (36.4%)	*n* = 4288 (27.5%)	*n* = 3680 (23.6%)	*n* = 1943 (12.5%)
Family physician				
≥1 visits	76.6	88.3	81.5	73.0
≥8 visits	10.4	15.7	21.0	14.3
Cognition specialist visits (≥1)	15.5	51.9	22.8	24.1
Other specialists visits (≥1)	56.2	98.2	80.6	73.4
ED visits				
≥1 visits	23.7	53.9	98.7	95.3
≥4 visits	0.5	5.1	30.0	29.1
Days in hospital (≥1)	1.3	16.8	96.3	67.3
30‐Day readmissions (yes)	0	0	28.1	27.6
Days In Alc (≥1)	0.4	0.4	1.2	46.6
LTCF admission (yes)	4.1	0.2	0.9	99.8
Death (yes)	4.2	0.0	26.1	Censored^†^

*Note*: Probabilities (%) for the final model can be interpreted as follows: probability for an individual to endorse an indicator, based on belonging to a given class. For example, an individual from group 1 has a 76.6% probability of presenting with ≥1 family physician visits; an individual from group 2 has a 98.2% probability of presenting with ≥1 other specialists visits; an individual in group 3 has a 96.3% probability of presenting with ≥1 hospital days; and an individual from group 4 has a 99.8% probability of being admitted in LTC.

Abbreviations: Alc, alternate level of care; ED, emergency department; LTCF, long‐term care facility.

^†^ In group 4, mortality was excluded for all persons admitted to long‐term care in the latent class analyses, as healthcare utilization was not measured after institutionalization due to data limitations. Thus, all the reported variables (except mortality rate) were considered before LCTF admission.

**Table 3:** Characteristics of individuals by group

Variables	Group 1: low users	Group 2: ambulatory centric users	Group 3: high acute hospital users	Group 4: long‐term care destined users	*P*‐value*
	*n* = 5673 (36.4%)	*n* = 4288 (27.5%)	*n* = 3680 (23.6%)	*n* = 1943 (12.5%)	
Median age, years (range)	83 (65; 106)	78 (65; 99)	84 (65; 103)	85 (65; 102)	<0.001
Female, *n* (%)	3916 (69.0)	2165 (50.5)	2237 (60.8)	1125 (57.9)	<0.001
Cohort inclusion, *n* (%)					
H: Hospitalization	1070 (18.9)	486 (11.3)	1217 (33.1)	419 (21.6)	<0.001
M: Physician visits	2247 (39.6)	2524 (58.9)	1531 (41.6)	1238 (63.7)	
R: Prescription profile	2356 (41.5)	1278 (29.8)	932 (25.3)	286 (14.7)	
Psychological comorbidity, *n* (%)					
Mood disorder	3270 (57.6)	2761 (64.4)	2391 (65.0)	1230 (63.3)	<0.001
Physical comorbidities, *n* (%)					
Chronic pulmonary disease	236 (4.2)	335 (7.8)	517 (14.0)	203 (10.4)	<0.001
Congestive heart failure	136 (2.4)	168 (3.9)	450 (12.2)	172 (8.9)	<0.001
Cardiac arrythmias	484 (8.5)	623 (14.5)	811 (22.0)	399 (20.5)	<0.001
Diabetes	666 (11.7)	666 (15.5)	700 (19.0)	344 (17.7)	<0.001
Hypertension	1274 (22.5)	1076 (25.1)	1191 (32.4)	576 (29.6)	<0.001
Tumor	189 (3.3)	581 (13.5)	480 (13.0)	192 (9.9)	<0.001
Median no. medications (range)^†^	9 (0; 38)	11 (0; 40)	15 (0; 51)	12 (0; 50)	<0.001
Medication, *n* (%)					
Acetylcholinesterase inhibitors	3483 (61.4)	2650 (61.8)	1581 (43.0)	620 (31.9)	<0.001
Antidepressants	1833 (32.3)	1686 (39.3)	1595 (43.3)	825 (42.5)	<0.001
Antipsychotics	1143 (20.1)	862 (20.1)	1353 (36.8)	835 (43.0)	<0.001
Benzodiazepines	1533 (27.0)	1208 (28.2)	1429 (38.8)	653 (33.6)	0.005
Mortality, *n* (%)^‡^	276 (4.9)	0 (0.0)	936 (25.4)	399 (20.5)	<0.001

* Statistical significance (*P* ≤ 0.001).

^†^ Medications were computed during the follow‐up period (1 year after the incident dementia diagnosis), and were based on having a medication claim in the drug dataset, for patients covered by the drug insurance plan.

^‡^ Due to data limitations, the variables presented in this table (except mortality) were considered before long‐term care facility admission. This is particularly relevant for group 4, where 1942 persons out of 1943 were admitted.

The first group, Low Users (*n* = 5673; 36.4%), consisted mainly of individuals with minimal healthcare utilization, and the lowest proportion of all measured comorbidities. This group had the highest proportion of women (69.0%; Table 3). Their probability of dying in the year after the diagnosis was low (4.9%), and a significant proportion of this group (41.5%, Table 2) met the R criterion, having a prescription profile for dementia.

The second group, labeled as Ambulatory Care‐Centric Users (*n* = 4288; 27.5%), showed the highest probability of visiting specialists, particularly cognition specialists (51.9%; Table 2). This group had the lowest percentage of individuals meeting the H criterion (11.3%), related to receiving a first dementia diagnosis during hospitalization. It was also the youngest group (median age of 78 years, Table 3) and presents no death during the follow‐up period.

The third group, categorized as High Acute Hospital Users (*n* = 3680; 23.6%), had the highest proportion of diagnosis received during hospitalization (related to H criterion; 33.1%). This group showed a 30% probability of frequent ED use (i.e. ≥4 visits over the year after the diagnosis). They also showed the highest proportion of heart disease (34.2%, including congestive heart failure and cardiac arrythmias), chronic pulmonary disease (14%), benzodiazepine use (38.8%) and mortality rate (25.4%).

The last group is referred to as Long‐Term Care Destined Users (*n* = 1943; 12.5%). Individuals mainly received their first dementia diagnosis during a physician visit, entering the cohort based on the M criterion (63.7%; Table 3). They had the highest probability of delayed discharge (46.6% probabilities of spending ≥1 days in alternate level of care; Table 2) and the highest proportion of antipsychotics claims (43%; Table 3). This group also had the highest probabilities of LTCF admission (99.8%), and a mortality rate of 20.5%.

## Discussion

Using LCA models, the present study identified four distinctive groups of persons with incident dementia, highlighting the heterogeneity of their healthcare use: (i) Low Users; (ii) Ambulatory Centric Users; (iii) High Acute Hospital Users; and (iv) Long‐Term Care Destined Users. Groups of low users consistently emerged as distinct profiles in research focused on healthcare use of PLWD,^15^, ^33^, ^34^, ^35^ suggesting low healthcare needs in a subset of PLWD.^15^, ^33^ Also, Low Users mainly entered the cohort through the R criterion, related to prescription. This observation suggests that their medication profiles align with early‐stage dementia, often prescribed as a symptomatic treatment against further cognitive decline.^36^ Low Users might not have progressed to a severe stage of dementia associated with higher healthcare use, during the follow‐up period.^37^ However, as we cannot conclude on the adequacy of care, it is essential to recognize that some in this group might face barriers to receiving appropriate care, resulting in unmet needs.^38^, ^39^ In this group, there was a high proportion of women. Older women, in particular, are less likely to have spousal caregivers, owing to women's longer life expectancy and common age disparities between married partners.^40^ This implies that women might not have their care coordinated by caregivers in a manner that encourages them to seek and access all necessary healthcare services.

The Ambulatory Care‐Centric Users' higher use of primary care, cognition and non‐cognition specialists implies that these patients benefit from comprehensive and ongoing healthcare management in the community. Older men are more likely to have a spousal caregiver than women^40^ who aids in the coordination of more suitable care than in the Low Users group, perhaps explaining, at least partly, the differing sex proportions observed between the Low Users and Ambulatory‐Centric Users groups.^41^, ^42^ Also, given the younger mean age of this group, combined with their higher visit frequency to family physicians and cognition specialists, we might speculate that dementia could have been detected earlier in these individuals. Primary care physicians play a crucial role in early dementia detection, yet the prevalence of undetected dementia lies at approximately 60% in the community.^43^

The High Acute Hospital Users had the highest proportion of comorbidities and mortality. These characteristics provide partial insight into their ED and hospital use, as healthcare needs for PLWD are known to increase with higher comorbidities.^37^, ^44^ Individuals in this group received a dementia diagnosis during hospitalization in higher proportions than what is typically reported in the general population PLWD, which is approximately 25%.^45^, ^46^ Diagnosis of dementia in the hospital setting is not consistent with best practice, as it can prolong stays and not lead to primary care follow up or specialist referrals.^47^ This is partly explained by a lack of communication between care settings and a complicated discharge process.^48^ The higher probability of 30‐day readmissions in this group might be related to a suboptimal discharge process and follow up,^49^ and is also associated with higher levels of comorbidity.^50^, ^51^

The Long‐Term Care Destined Users had the highest probability of LTCF admission. Among PLWD, LTCF admission is notably associated with older age, not being married, more severe dementia, behavioral and psychological symptoms of dementia, functional impairment and caregiver burden.^52^ They were the highest users of antipsychotics before LTCF admission, consistent with the fact that many antipsychotic prescriptions among PLWD in LTCF were initiated before admission, and for reasons related to behavioral and psychological symptoms of dementia.^53^ The higher probability of 30‐day readmission in this group also raises questions about community service provisions and the timing for LTCF placement,^54^ an area that should be further explored.

The strengths of the current study include the use of a large province‐wide administrative database, a near‐complete cohort of Quebecer persons with incident dementia. Classification methods are strategies of growing interest to guide clinical practice, especially when based on large medical/administrative datasets, allowing for generalization to the whole population. Regarding limitations, persons who were misdiagnosed, undiagnosed or unknown to the healthcare system for dementia were not included in the cohort. Administrative data do not offer access to healthcare dispensed by salaried physicians, nurses, social workers, allied healthcare care workers, non‐pharmacological treatments or informal caregiving. LCA classification is subject to a degree of subjectivity, results might not necessarily generalize and would benefit from replication, as well as pairwise comparisons between the groups.^25^

Health and social care professionals must be aware of the range of complex and heterogeneous needs of their patients to support their care.^55^, ^56^ Although the current study included a wide variety of services for dementia (ambulatory care, hospital‐based care, LTCF and medications), preventative approaches might be better suited to reduce unnecessary or avoidable healthcare use through the course of the illness. For example, an emphasis on early assessment and the provision of tailored services, such as home care, is essential to prevent patients entering unfavorable profiles. Equally important is improving dementia diagnosis in primary and early care to facilitate care planning and prevent situations, such as delayed discharge.

In addition to the present research, future studies should further examine the social and medical needs of PLWD, such as their living arrangements, socioeconomic status and the use of home care. In addition, longitudinal studies that not only examine care patterns, but also the trajectories of care, can help us understand how patients' needs change through time. This understanding can lead to the development of targeted interventions that address individuals' needs as their condition progresses.

In conclusion, the present study provides context on the heterogeneity in healthcare use after a first dementia diagnosis. Identifying subgroups with defined characteristics sets a precedent for interventions targeted to distinct needs rather than one‐size‐fits‐all solutions.

## Funding information

ID received a postdoctoral scholarship from the Alzheimer Society Research Program (ASRP) of Canada. Caroline Sirois received a FRQS Junior 2 Research Scholar Award. The funding sources were not involved in the preparation and submission of this manuscript.

## Disclosure statement

The authors declare no conflict of interest.

## Ethics statement

This study is part of the Institut national de santé publique du Québec's ongoing chronic disease surveillance mandate, approved by the Provincial Public Health Ethics Committee, which allows surveillance activities without participant consent. It was also approved by the McGill Faculty of Medicine Institutional Review Board.

## Patient consent statement

Informed consent was not necessary for this study, as this study used anonymized data from a health administrative database.

## Data Availability

The data that support the findings of this study are available from INSPQ. Restrictions apply to the availability of these data, which were used under license for this study. Data are available from the author(s) with the permission of INSPQ.

## Appendix I

**Table A1:** Statistical indicators for models with increasing numbers of latent classes.

	1‐Class solution	2‐Class solution	3‐Class solution	4‐Class solution	5‐Class solution
AIC	356 671.1	343 796.2	340 999.9	340 251	353 716.7
BIC	364 240.9	358 989.4	363 816.4	370 690.9	391 780.0

Abbreviations: AIC, Akaike's Information Criterion; BIC, Bayesian Information criterion.
